# An injured pachypleurosaur (Diapsida: Sauropterygia) from the Middle Triassic Luoping Biota indicating predation pressure in the Mesozoic

**DOI:** 10.1038/s41598-021-01309-z

**Published:** 2021-11-08

**Authors:** Qiling Liu, Tinglu Yang, Long Cheng, Michael J. Benton, Benjamin C. Moon, Chunbo Yan, Zhihui An, Li Tian

**Affiliations:** 1 State Key Laboratory of Biogeology and Environmental Geology, China University of Geosciences, Wuhan, 430078 People’s Republic of China; 2 School of Earth Science, East China University of Technology, Nanchang, 330013 Jiangxi Province People’s Republic of China; 3 Hubei Key Laboratory of Paleontology and Geological Environment Evolution, Wuhan Centre of China Geological Survey, Wuhan, 430023 Hubei People’s Republic of China; 4 School of Earth Sciences, Life Sciences Building, Tyndall Avenue, University of Bristol, Bristol, BS8 1TQ UK

**Keywords:** Palaeontology, Palaeoecology

## Abstract

The Middle Triassic Luoping Biota in south-west China represents the inception of modern marine ecosystems, with abundant and diverse arthropods, fishes and marine reptiles, indicating recovery from the Permian–Triassic mass extinction. Here we report a new specimen of the predatory marine reptile *Diandongosaurus*, based on a nearly complete skeleton. The specimen is larger than most other known pachypleurosaurs, and the body shape, caniniform teeth, clavicle with anterior process, and flat distal end of the anterior caudal ribs show its affinities with *Diandongosaurus acutidentatus*, while the new specimen is approximately three times larger than the holotype. The morphological characters indicate that the new specimen is an adult of *D. acutidentatus*, allowing for ontogenetic variation. The fang-like teeth and large body size confirm it was a predator, but the amputated hind limb on the right side indicate itself had been predated by an unknown hunter. Predation on such a large predator reveals that predation pressure in the early Mesozoic was intensive, a possible early hint of the Mesozoic Marine Revolution.

## Introduction

The first large marine reptiles evolved in the Early and Middle Triassic^1,2^, forming part of the ‘modern-style’ marine ecosystems that emerged as life recovered from the Permian–Triassic mass extinction (PTME)^3^. This was also arguably the time when the Mesozoic Marine Revolution (MMR) began^4–6^, a long-established ecological shift towards higher productivity of marine ecosystems associated with arms races between predators and prey^7^. New groups of molluscs and arthropods provided food for predatory gastropods, crustaceans, fishes and reptiles. Here we report a relatively large predatory pachypleurosaur that had been preyed upon by an even larger predator, providing direct evidence for an MMR arms race in action.

The Triassic marine recovery is well documented in southern China, by a sequence of marine faunas, including the Nanzhang-Yuan’an, Chaohu, Panxian, Luoping, Xingyi and Guanling faunas. In particular, the Luoping Biota of the Middle Triassic, yielding nearly 20,000 macrofossils, provides extraordinary records of very early marine reptiles^8,9^. In contrast to modern marine ecosystems, hypercarnivores that fed on other tetrapods were common in Mesozoic oceans, confirming a different trophic structure at that time^1^.

Here we report and describe a new large marine pachypleurosaur species from the Luoping Biota, decipher its role in eosauropterygian evolution, and its ecological implications in the recovery of ecosystems and megafaunal predation in the early Mesozoic oceans.

## Results

### Geological background

The Luoping Biota from quarries near Daaozi Village, Luoping County, Yunnan Province, China, includes diverse arthropods, conodonts, foraminifers, molluscs, echinoderms, brachiopods, fishes, marine reptiles, plants, and trace fossils^8,10–13^. The fossil beds occur in Member II of the Guanling Formation which in the Daaozi section comprises approximately 16 m of dark-coloured micritic limestone, thin to moderately thickly bedded, indicating a semi-enclosed intraplatform setting^10,11^. The co-occurring conodont assemblages, primarily consisting of *Cratognathodus* sp. and *Nicoraella kockeli*, indicate that the Luoping Biota belongs to the Pelsonian Substage of the middle Anisian, and the U–Pb age, which is 246.6 ± 1.4 Ma, of the volcanic tuff at the bottom of Member I confirms this age^10,14^.

### Systematic palaeontology

Superorder **Sauropterygia** Owen, 1860^15^.

Order **Eosauropterygia** Rieppel, 1994^16^.

Family **Incertae Sedis**.

Genus ***Diandongosaurus*** Shang, Wu & Li, 2011.

### Type species

*Diandongosaurus acutidentatus* Shang, Wu & Li, 2011.

### Revised diagnosis

Small-to-medium-sized eosauropterygian with the following unique combination of characters: premaxilla with long, fang-shaped teeth; maxilla with single enlarged fang alongside smaller teeth; parietal foramen about level with anterior margin of supratemporal fenestra; supratemporal smaller than orbit; interorbital bridge broad; frontal excluded from orbit; posterolateral processes of frontal extending over anterior margin of supratemporal fenestra; postorbital excluded from infratemporal fenestra by contact between jugal and squamosal; ectopterygoid present; vertebral column consisting of about 38 presacral, 3 sacral, and more than 30 caudal vertebrae; anterior caudal ribs elongate without tapering distal end; clavicle with distinct anterior processes laterally; entepicondylar foramen absent; acetabular process of pubis strongly offset from the main body.

*Diandongosaurus* cf. *acutidentatus.*

### Material

**Figure 1:**
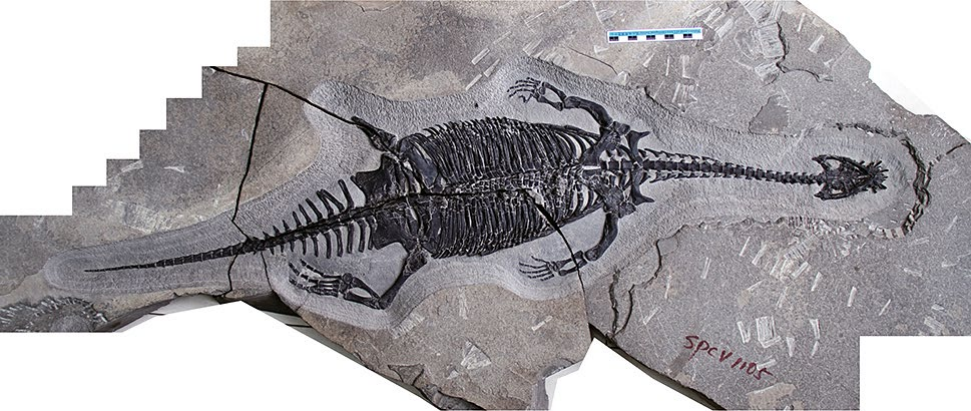
Full skeleton of WIGM SPC V 1105, viewed from above. Note the missing left foot. Scale bar = 10 cm.

WIGM SPC V 1105, a nearly complete skeleton exposed ventrally (Fig. 1).

### Locality and horizon

Daaozi Village, Luoping County, Yunnan Province, China; Member II of the Guanling Formation, Anisian, Middle Triassic.

### Description

WIGM SPC V 1105 is a large pachypleurosaur with a length of 88.6 cm from the tip of the snout to the end of the caudal vertebral column (Fig. 1). The specimen is exposed in ventral view, with the cranium exposed both ventrally and dorsally. In the holotype, the cranium comprises 7.8% of the total length, neck 22.9%, trunk 32.4%, and tail 36.9% (Table 1).

**Table 1:** Selected measurements (in mm) of WIGM SPC V 1105.

**Skull and mandible**	
Length from tip of snout to end of skull table	69
Length from tip of snout to anterior margin of external naris	13
Length from tip of snout to anterior margin of orbit	29
Length from tip of snout to anterior margin of supratemporal fenestra	44
Length between naris and orbit	7
Maximum length of external naris	9
Maximum length of orbit	13
Maximum length of supratemporal fenestra	10
Maximum length of parietal foramen	3
Width between external naris	3
Width between orbits	11
Length of retroarticular process	11
Width of retroarticular process	4
**Postcranial skeleton**	
Length of preserved postcranial skeleton	816
Length of atlas centrum	5
Length of axis centrum	10
Length of right humerus	56
Proximal width of right humerus	12
Distal width of right humerus	12
Minimal width of right humerus	10
Length of right ulna	30
Length of right radius	30
Length of right metacarpal 3	13
Maximum width of right intermedium	9
Maximum width of right ulnare	8
Length of left femur	74
Proximal width of left femur	21
Distal length of left femur	13
Minimal width of left femur	8
Length of left tibia	41
Length of left metatarsal 3	23
Maximum width of left calcaneum	12
Maximum width of left astragalus	15

### Skull

**Figure 2:**
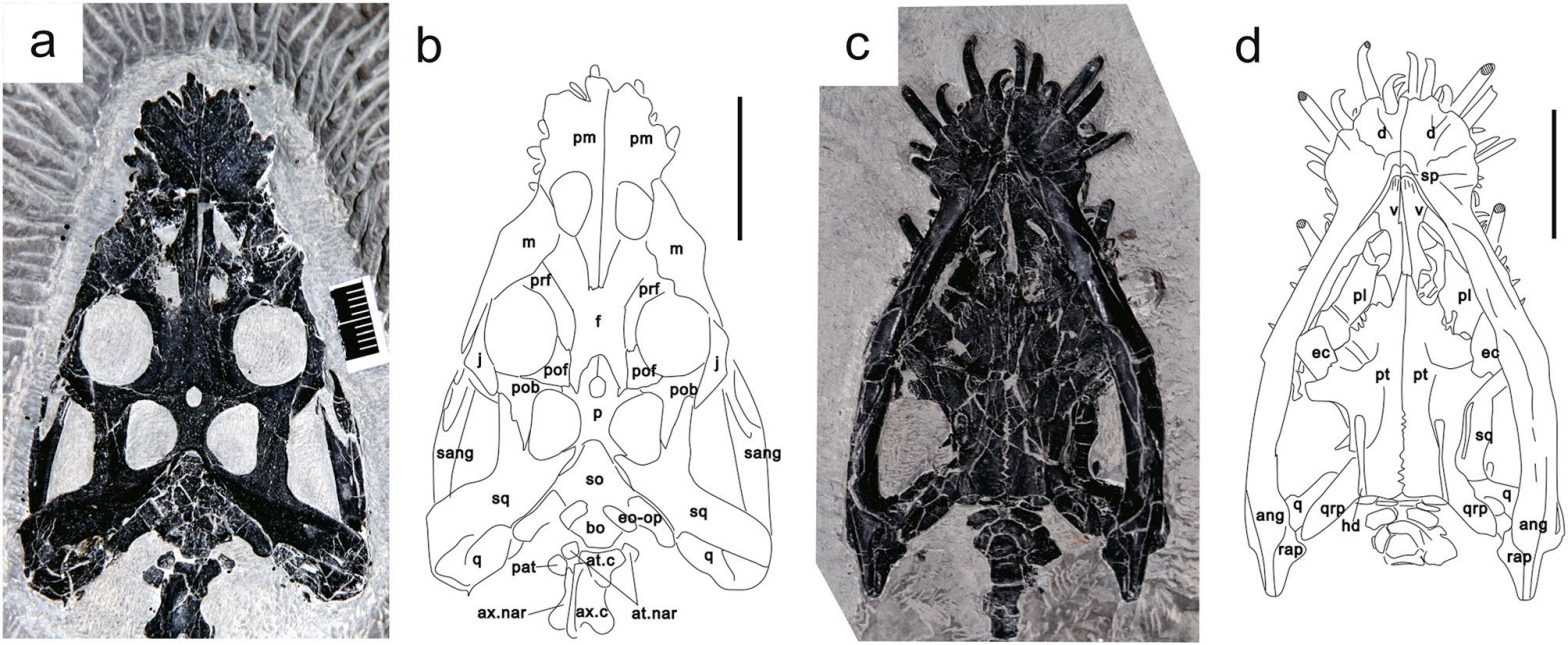
Photograph and interpretative drawing of the skull of WIGM SPC V 1105. (**a**, **b**) In dorsal view; (**c**, **d**) In ventral view. *ang.* Angular, *at.c* atlantal centrum, *at.nar* atlantal neural arch, *ax.c* axial centrum, *ax.nar* axial neural arch, *bo* basioccipital, *d* dentary, *ec* ectopterygoid, *eo-op* exoccipital-opisthotic, *f* frontal, *hd* hyoid, *j* jugal, *m* maxilla, *n* nasal, *p* parietal, *pat* proatlas, *pl* palatine, *pm* premaxilla, *pob* postorbital, *pof* postfrontal, *prf* prefrontal, *pt* pterygoid, *q* quadrate, *qrp* quadrate ramus of pterygoid, *rap* retroarticular process, *sang* surangular, *so* supraoccipital, *sp* splenial, *sq* squamosal, *vo* vomer. Scale divisions in (**a**) = 1 mm. Scale bar in (**b**–**d**) = 2 cm. The figure is generated using CorelDRAW X7 (https://www.coreldraw.com/en/pages/coreldraw-x7/).

The skull of WIGM SPC V 1105 is exposed in both dorsal and ventral views and is dorsoventrally compressed (Fig. 2). The external naris and the supratemporal fenestra are oval-shaped, while the orbit is nearly circular.

In dorsal view (Fig. 2a,b), the premaxillary portion of the rostrum protrudes, defined by snout constriction at the anterior maxilla, different from the reported specimens of *D. acutidentatus*^17,18^. The premaxilla forms the anterior and the medial margins of the external naris. The nasal process extends and narrows posteriorly alongside the nasal posteromedially, reaching the anterior margin of the orbit, and contacting the anterior frontal with a cuspidal border line. The premaxilla contacts the maxilla lateral to the external naris.

The maxilla is elongate, with a laterally broad anterior portion and tapering posterior process. Its anteromedial margin forms the posterolateral border of the external naris and is overlapped by the posterior premaxilla laterally. The anterior snout constriction is mostly defined by strong medial curvature of the anterolateral maxilla margin. Medially the maxilla contacts the nasal immediately posterior to the external naris, and the prefrontal posterior to that; the nasal contact is likely the longer. Posteriorly, the maxilla borders the anterolateral margin of the orbit. The posterior process of the maxilla contacts the jugal lateral to the orbit. The nasals are broken. They are separated medially by the premaxilla and make a small contribution to the posterior external naris. The external naris is subcircular.

The prefrontal is an arch-shaped bone, fused with the lacrimal. Its dorsal portion expands posteriorly, with its ventral portion forming the anterodorsal margin of the orbit. Posteriorly, the prefrontal overlaps the postfrontal obliquely at the midpoint of the dorsal border of the orbit. The postfrontal is a small trapezoid-shaped bone that forms the posterodorsal margin of the orbit, and is more extensive than in *Dianopachysaurus dingi*^19^. Posteriorly, it meets the postorbital anterior to the supratemporal fenestra and has a small medial contact with the parietal, separating the postfrontal from the supratemporal fenestra. Both the prefrontal and postfrontal contact the frontal dorsally, preventing it from entering the orbit.

The frontals are fused medially into a butterfly shape in dorsal view, expanding obliquely in four directions. Anteriorly the contacts with the nasals are uncertain but were likely to have been broad. The median contact with the premaxilla is narrow and irregular. The frontal meets the prefrontal and the postfrontal laterally along the arc of the dorsal orbital margin, preventing it from entering the orbit, as in *Diandongsosaurus acutidentatus*^17^, but unlike both *Keichousaurus hui* and *Dianopachysaurus dingi*^19,20^. The frontal does not enter the supratemporal fenestra either, being narrowly excluded by the parietal and the postorbital as in *D. acutidentatus*^17^. In *Dianopachysaurus dingi*^19^, contact between the postfrontal and parietal excludes the frontal from the supratemporal fenestra. Posteriorly, the frontal expands slightly, laterally towards the supratemporal fenestrae, and diverges into a narrow fork around the anterior processes of the parietals, separating them from the postfrontal.

The parietals are partly fused, showing a suture only anterior to the pineal foramen. The anterior processes insert between the posterior frontal margins with an arch-shaped border. Laterally, the parietal extends a short process to meet the postorbital in a narrow contact at the anterior margin of the supratemporal fenestra, posterior to the postfrontal. This differs from *K. hui* and *Dianopachysaurus dingi*^19,20^, in which the parietal contacts the postfrontal anterolaterally. The bone forms the medial margin of the supratemporal fenestra. The narrow posterolateral processes are inserted by the dorsal processes of the squamosal. The pineal foramen is sub-circular and aligns with the anterior margin of the supratemporal fenestra, more anterior than in *K. hui*^20^ and not elongate as in *Dianopachysaurus dingi*^19^.

The postorbital is roughly triradiate, developing three processes: anteroventral, anteromedial, and posterior. The anteroventral process outlines the posterior border of the orbital, overlapped by the jugal laterally. The narrow anteromedial process extends dorsally, forming the anterior margin of the supratemporal fenestra, and meeting the postfrontal and the parietal anterior to the supratemporal fenestra, unlike in the reported specimens of *D. acutidentatus*, *K. hui*, and *Dianopachysaurus dingi*^17,19,20^, and more like nothosaurs^21,22^. It is broadly overlapped by the postfrontal. The posterior process is triangular and extends nearly to the posterolateral margin of the supratemporal fenestra, forming the border of most of its lateral portion. Posteriorly, the tip of the process inserts into the squamosal.

The jugal is boomerang-shaped, forming most of the lateral border of the orbit. It contacts the maxilla at the anteroventral margin of the orbital, dorsally overlapping it. Posteriorly, the jugal forms the anterior border of the infratemporal fenestra. Its posterior process is anteroposteriorly broad and extends dorsally, overlapping the postorbital at the posteroventral margin of the orbital. As in *D. acutidentatus*^17^, the posterior process of the jugal has a small contact dorsally with the anterior process of the squamosal.

The squamosal is a large bone expanded in four directions. The anterior process forms most of the upper temporal bar, extending anterior to the level of the anterior margin of the supratemporal fenestra and partially overlapped medially by the postorbital, except where the squamosal holds the posteriormost point of the postorbital. Anteriormost on the squamosal, there is a small lateral contact with the posterior process of the jugal. The medial process of the squamosal forms almost the whole posterior margin of the supratemporal fenestra, inserting into the posterolateral process of the parietal medially. The posterolateral descending process is robust and expands ventrally, forming a sheet at the posterior margin of the cranium and contacting the lateral portion of the quadrate on its posteromedial face. However, the posterior process, the shortest of these four processes, is not as obvious as in the reported specimens of *Dianopachysaurus. acutidentatus* or *K. hui*^17,20^. The supratemporal fenestra is rounded and smaller than the orbit, with a straighter lateral margin. It is less elongate than in *Dianopachysaurus dingi* and *K. hui*^19,20^.

The quadratojugal is not exposed. The supraoccipital is a rhomboid bone inserted ventral to the parietal but is substantially broken; it forms the dorsal margin of the foramen magnum. The exoccipital-opisthotic forms the lateral margin of the foramen magnum, while the basioccipital forms the ventral; these elements are also broken.

In ventral view (Fig. 2c,d), the internal choana is roughly circular. The vomer is a long bone with a bifurcating posterior portion along the midline of the palate and forms the medial margin of the internal choana. Anteriorly, the bone meets the palatal portion of the premaxilla and contacts the maxilla anterolaterally. Posteriorly, the posteromedial processes of the two vomers are separated by the anterior process of the pterygoid and the posterior contact with the palatine is small, as in *D. acutidentatu*s^18,22^ but unlike in *K. hui*^20^.

The palatine is a strap-like bone. It forms the posterolateral margin of the internal choanae. Anterolaterally, it contacts the maxilla, and meets the vomer on its medial side. Posteromedially, there is a highly irregular, oblique suture line between the palatine and the pterygoid.

The pterygoid is one of the largest bones of the skull, forming most of the palate posteriorly. The two pterygoids are fused along the midline leaving a straight groove anteriorly that becomes more irregular posteriorly. Unlike *D. acutidentatus*, it has neither central opening, nor posterior vacuity^18^. The tapering anterior process of the pterygoid inserts between the two vomers, whereas it is overlapped in *K. hui*^20^, and anterolaterally the pterygoid has a large oblique contact with the palatine. Laterally, the transverse process of the pterygoid expands ventral and posterior to the posterior margin of the ectopterygoid. The pterygoid forms almost the entirety of the subtemporal fenestra margin anteriorly, medially, and posteriorly. The elongate quadrate ramus of the pterygoid extends posterolaterally to the posterior margin of the quadrate, making a long contact with the pterygoid ramus of the quadrate.

The ectopterygoid is roughly a small square bone, suturing to the transverse process of the pterygoid. It is not as prominent as in nothosaurs (e.g. *Nothosaurus*^21^, *Lariosaurus*^22^), but is relatively larger than in the reported specimens of *D. acutidentatu*s^18,23^, whereas the presence of an ectopterygoid is uncertain in *K. hui* and *Dianopachysaurus dingi*^19,20^. The ectopterygoid contacts the palatine anteriorly, excluding the palatine from the subtemporal fenestra. Posteriorly it makes a small contribution to the subtemporal fenestra margin lateral to the transverse process of the pterygoid. The quadrate is exposed partly, contacting the quadrate ramus of the pterygoid with its pterygoid ramus. Two rod-like hyoids are ossified and well preserved, lying beneath the pterygoid. They are elongate and slightly expanded at each end.

### Mandible

The mandible is exposed mainly in ventral view and partly in dorsal (Fig. 2). The dentary is a long bone, occupying over one-half of the ramus as a counterpart to the premaxilla, with a laterally broader symphyseal portion than in *D. acutidentatus* or *K. hui*^18,20,23^. The surangular is partly exposed in dorsal view along the dorsal margin of the mandible, extending ventral to the squamosal. The angular is a long strap-shaped bone that meets the dentary anteriorly and the retroarticular process posteriorly. The articular is sutured dorsal to the angular, with a distinct retroarticular process that extends posteriorly with a tapering end.

### Dentition

In ventral view (Fig. 2c,d), nine premaxillary teeth and seven lower teeth are visible, which are procumbent, fang-like and with apicobasal striations. The 2nd and 3rd right and the 1st, 3rd and 5th left premaxillary teeth are fully grown, elongate and less curved compared to the other teeth. However, the reported specimens of *D. acutidentatus* and the nothosauroids *Lariosaurus* and *Nothosaurus* carry five teeth on each premaxilla^17^. The space between the 2nd and 3rd right premaxillary teeth suggests that there might be one or two missing teeth. There is one fang-like tooth on each maxilla, surrounded by small tapering teeth, and there are five to six corresponding teeth in the lower jaw. The caniniform teeth also have apicobasal striations like the premaxillary teeth. The row of dentary teeth is restricted to a level anterior to the posterior margin of the orbit.

### Vertebrae and ribs

There are 38 presacral vertebrae, 3 sacral and 33 caudal (Fig. 1); these counts are roughly the same in coeval Eosauropterygia^19,24,25^. The atlas and axis are dorsally exposed (Fig. 2a,b). The atlas leans anteriorly, and its neural spine does not meet its counterpart. The proatlas is a pentagonal bone, disarticulated from the atlas. The axis has been rotated laterally, but still articulates with the atlas.

There are 19 cervical vertebrae, compared to 20/21 in *Dianopachysaurus dingi*^19^. The centra cylinders are rhomboidal in ventral view, increase in length posteriorly and the vertebrae articulate with one another compactly. The parapophyseal articulation on the cervical rib (CR), visible in ventral view, is robust and offset about 90° from the long axis of the rib, defined between the main body and a prominent anterior process. These posterior and anterior extensions are approximately equal in length until about CR14, where the posterior extension starts to lengthen strongly. The anterior process becomes strongly reduced from CR16 onwards.

There are approximately 19 thoracolumbar vertebrae, most of which are covered by the gastralia (18 in *Dianmeisaurus gracilis*^25^); the count estimated from two gastralial rows corresponding to one vertebra. The intercentral articulation is less compact than in the cervical vertebrae. The transverse processes face posteriorly. The dorsal ribs are single-headed arch-shaped bones with slightly expanded proximal flat ends, but otherwise retain constant diameter along their whole length, ending distally in a flattened stub. Dorsal ribs DR1–6 are exposed ventrally, while the rest are mostly overlain by the gastralia. There are 24 rows of gastralia, suggesting 12 more dorsal vertebrae covered, each gastralium consisting of one medial element and four lateral elements (Fig. 4a).

**Figure 3:**
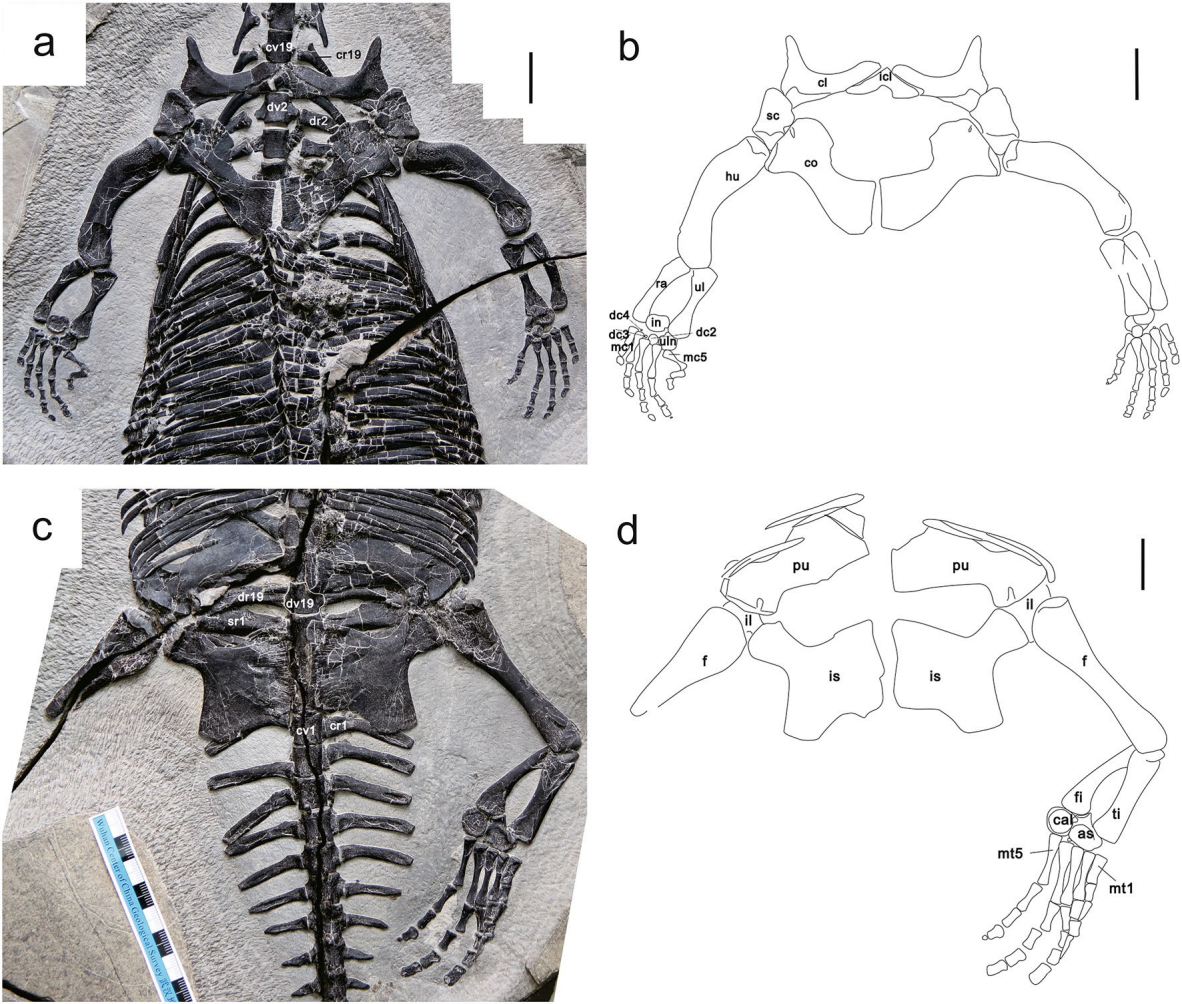
Photographs and interpretative drawings of the pectoral girdle, forelimb, pelvic girdle and hindlimb of WIGM SPC V 1105 in ventral view. (**a**, **b**) Pectoral girdle and forelimb. (**c**, **d**) Pelvic girdle and hindlimb. *as* astragalus, *cal* calcaneum, *cl* clavicle, *co* coracoid, *cr1* caudal rib 1, *cr19* cervical rib 19, *cv1* caudal vertebra 1, *cv19* cervical vertebra 19, *dc2* distal carpal 2, *dc3* distal carpal 3, *dc4* distal carpal 4, *dr2* dorsal rib 2, *dv2* dorsal vertebra 2, *dr19* dorsal rib 19, *dv19* dorsal vertebra 19, *f* femur, *fi* fibular, *hu* humerus, *icl* interclavicle, *il* Ilium, *in* intermedium, *is* ischium, *mc1* metacarpal 1, *mc5* metacarpal 5, *mt1* metatarsal 1, *mt5* metatarsal 5, *pu* pubis, *ra* radius, *sc* scapula, *sr1* sacral rib 1, *ti* tibia, *ul* ulna, *uln* ulnare. Scale bar in (**a**, **b**, **d**) = 2 cm. Scale divisions in (**a**) = 1 mm. The figure is generated using CorelDRAW X7 (https://www.coreldraw.com/en/pages/coreldraw-x7/).

**Figure 4:**
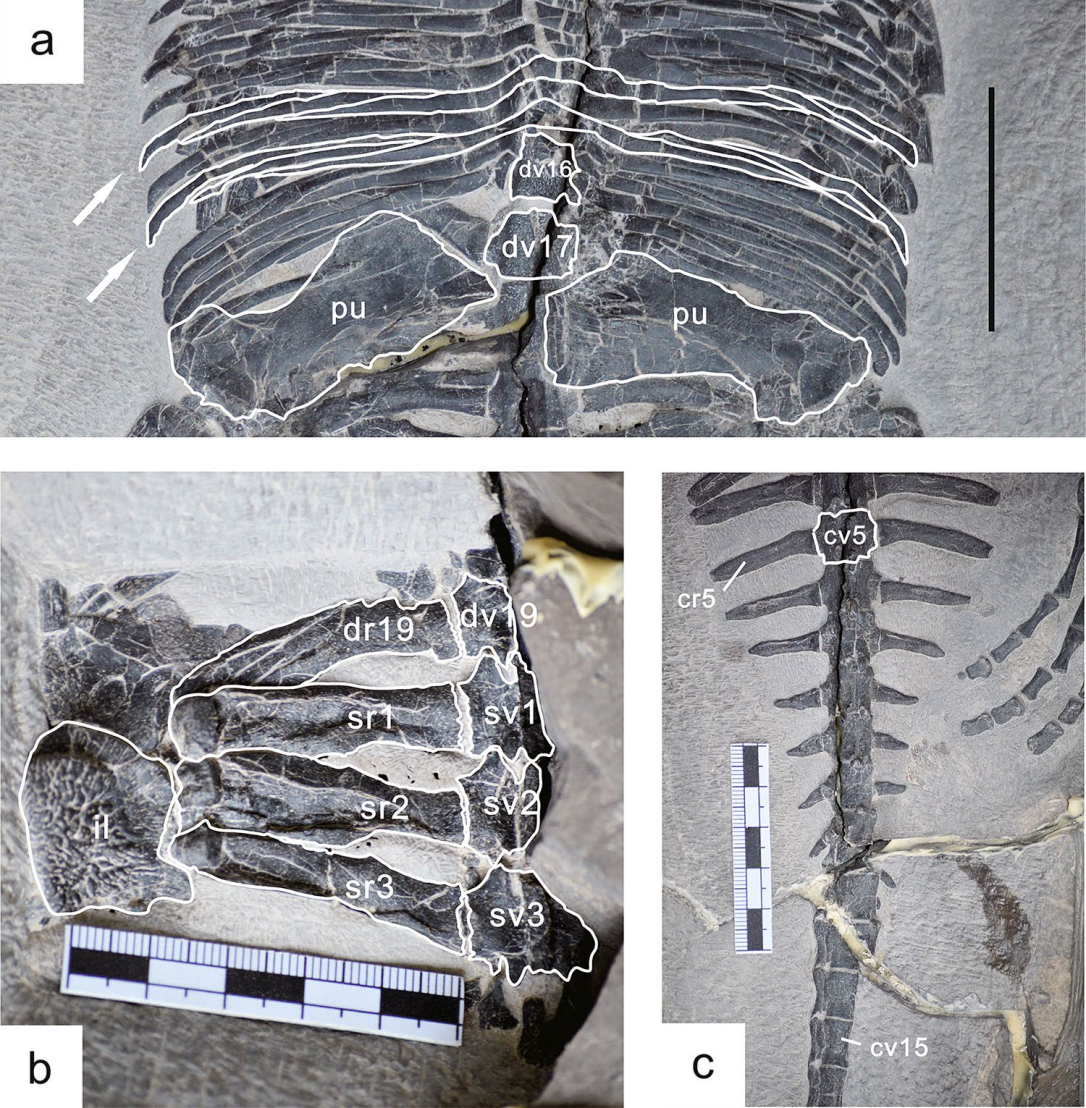
Selected postcranial parts of WIGM SPC V 1105. (**a**) gastralia near the sacral region in ventral view, the arrow indicating each gastralium consists of one medial element and four lateral elements; (**b**) sacral region in dorsal view; (**c**) part of the caudal region in ventral view. *cr5* caudal rib 5, *cv5* caudal vertebra 5, *cv15* caudal vertebra 15, *dr19* dorsal rib 19, *dv16* dorsal vertebra 16, *dv17* dorsal vertebra 17, *dv19* dorsal vertebra 19, *il* ilium, *pu* pubis, *sr1* sacral rib 1, *sr2* sacral rib 2, *sr3* sacral rib 3, *sv1* sacral vertebra 1, *sv2* sacral vertebra 2, *sv3* sacral vertebra 3. Scale bar = 5 cm. The figure is generated using CorelDRAW X7 (https://www.coreldraw.com/en/pages/coreldraw-x7/).

Three sacral vertebrae can be recognized in dorsal view (Fig. 4b), the same as in *Dianmeisaurus gracilis*, *Dianmeisaurus dingi* and *K. hui*^19,24,26^. The sacral ribs are elongate and cylindrical with thickened distal ends, and closely articulate with the centrum and possibly overlap the rib posterior to each proximally. Distally the sacral rib is expanded posteriorly into a small triangular process that overlaps the next sacral rib posteriorly. Sacral ribs SR2 and SR3 likely articulate with the ilium, while the others are overlain by pubis and ischium (Fig. 3c,d).

There are 33 rhomboidal caudal vertebrae that decrease in size gradually towards the posterior end of the tail. Caudal vertebrae CV13–21 have strap-shaped neural spines. Caudal ribs are present in CV1–11. They are flat, arch-shaped bones directed slightly posteriorly. The size of the ribs remains roughly the same from CR1–5, but this decreases suddenly from CR6–11 (Fig. 4c). The distal ends of CR3–8 are flat, while more posterior ribs have pointed ends.

### Pectoral girdle and forelimb

The pectoral girdle is exposed in ventral view (Fig. 3a,b). The interclavicle is an arrowhead-shaped bone with a strongly concave posterior border and two posterolaterally directed lateral processes, unlike the more diamond shape of *D. gracilis*^24^. Its tip points anteriorly but does not reach the anterior margin of the pectoral girdle between the clavicles. The clavicle is an L-shaped, strap-like bone with a characteristic prominence anterolaterally, as in *D. acutidentatus* and larger than in *D. gracilis*^17,24^. The clavicle develops a tiny posterolateral process, overlying the dorsal surface of the scapula. The tapering medial process expands to meet its counterpart, forming the anterior margin of the pectoral girdle. The scapula is exposed in ventral view, so the dorsal blade is covered. In this view it is sub-rectangular, with a rounded anterior margin and two posterior facets for the clavicle and humerus, angled obliquely and separated by a small ridge. The coracoid is a strap-shaped bone with proximal and distal ends widened, and the largest element in the pectoral girdle. Its anteromedial margin is more strongly concave than the posteromedial margin. Proximally, the coracoid is flattened and meets the contralateral element in a straight median facet. Distally the coracoid is more robust and expanded anteriorly into a broad rounded process on the anterior margin. The distal margin is straight and articulates with the scapula anteriorly and has a smaller articulation with the humerus posteriorly on a smaller, triangular posterodistal process. There is a small foramen exposed near the anterodistal margin along the scapular facet, larger than in *Dianmeisaurus gracilis*^24^.

Both forelimbs are nearly complete, ventrally exposed, about 13.7% of the body length (Fig. 3a,b). The humerus is strongly curved (40°) and shorter than the femur (Table 1). The proximal articular surface is rounded, with a larger facet for the scapula than the coracoid, while the articular surface of the distal end is convex, contacting the radius and the ulna with two straight, oblique facets. These facets are more strongly offset than in *D. acutidentatus*^17^. There is no evidence for an entepicondylar foramen^20,24^. The ulna and the radius are nearly equal in length and relatively gracile compared to the humerus (Table 1). The two ends of the ulna are equally widened, while the ends of the radius expand less obviously and are directed slightly medially.

There are more than four elements in the carpus, all round and flat in ventral view. The intermedium is slightly larger than the ulnare (Table 1), unlike in *D. acutidentatus*^17^, and articulates mediodistally to the ulna, medially to the ulnare. Distal carpal 2 is the largest of the distal carpals and articulates distally between the intermedium and ulnare. Distal carpals 3 and 4 are present but extremely reduced. The metacarpals are elongate and strongly hourglass shaped. Metacarpal 1 is the shortest of the five while metacarpals 2–4 are almost equal in length, and metacarpal 5 is slightly shorter. All the digits are directed towards the ulnar side of the limb. The interosseous space between metacarpals 4 and 5 is the widest. The phalangeal elements are well preserved, but digit 5 of the right manus demonstrates unusual preservation, which will be discussed further in the Discussion. The ungual phalanges of digits 4 and 5 on the left are small and round, while the ungual phalanx of digit 5 on the right is missing. Given that, the forelimb is likely to have had a phalangeal formula of 2–3–4–4–3.

### Pelvic girdle and hindlimb

The pelvic girdle is exposed ventrally (Fig. 3c,d). The pubis is a large plate-like bone. Both the anterior and posterior margins of the bone are concave near the distal end (about one-third of the whole length), forming a ‘waisted’ shape that is narrower than in *Dianmeisaurus gracilis*^24^. The ischium is large and irregularly shaped. Medially it is expanded into a large, squared, plate-like portion that meets the contralateral element along a straight median symphysis. Anterodistally, the ischium is waisted, separating the large, robust anterodistal process with a broad, rounded end that contacts the distal pubis and ilium to form the acetabulum. The anterodistal process is narrower and more strongly offset from the main body than in *Dianmeisaurus gracilis*^24^. Posterodistally there is a further broad extension. The thyroid fenestra is large and rectangular and is bounded by the posterior pubis and anterior ischium on both sides. The ilium is covered by the pubis and the ischium in ventral view.

The left hindlimb is well preserved and exposed in ventral view (Fig. 3c,d), and the amputated right femur is discussed below. The femur is long and rounded with a slightly waisted epiphysis; it is larger and slenderer than the humerus (Table 1). The proximal end is wider than the distal but is damaged in this specimen. The tibia and the fibula are similarly elongate bones, with the tibia somewhat more robust but more similar in size than in the holotype of *D. acutidentatus*^17^. Both have slightly expanded proximal and distal ends, but the proximal end of the fibula is hidden beneath the distal femur. The stronger waist on the fibula gives it a more strongly curved appearance and creates a large interosseous fenestra.

The astragalus and calcaneum are the only elements of the tarsus. The astragalus is larger than the calcaneum and located between the distal tibia and fibula with a pointed proximal margin (Table 1). The facets of the astragalus contacting the tibia and the fibula are straight. The calcaneum is subcircular. Length increases from metatarsals 1–4, then decreases in metatarsal 5; metatarsal 1 is the shortest. All the metatarsals have an elongate hourglass shape. The pes is not so well preserved, as digits 1 and 2 are crushed together. The phalanges are less elongate than the metatarsals and shaped like waisted cylinders, except for the ungual phalanx of digit 5; consequently, there may be some missing ungual phalanges from the other digits. The pedal phalangeal formula cannot be determined due to the preservation.

### Phylogenetic analysis

**Figure 5:**
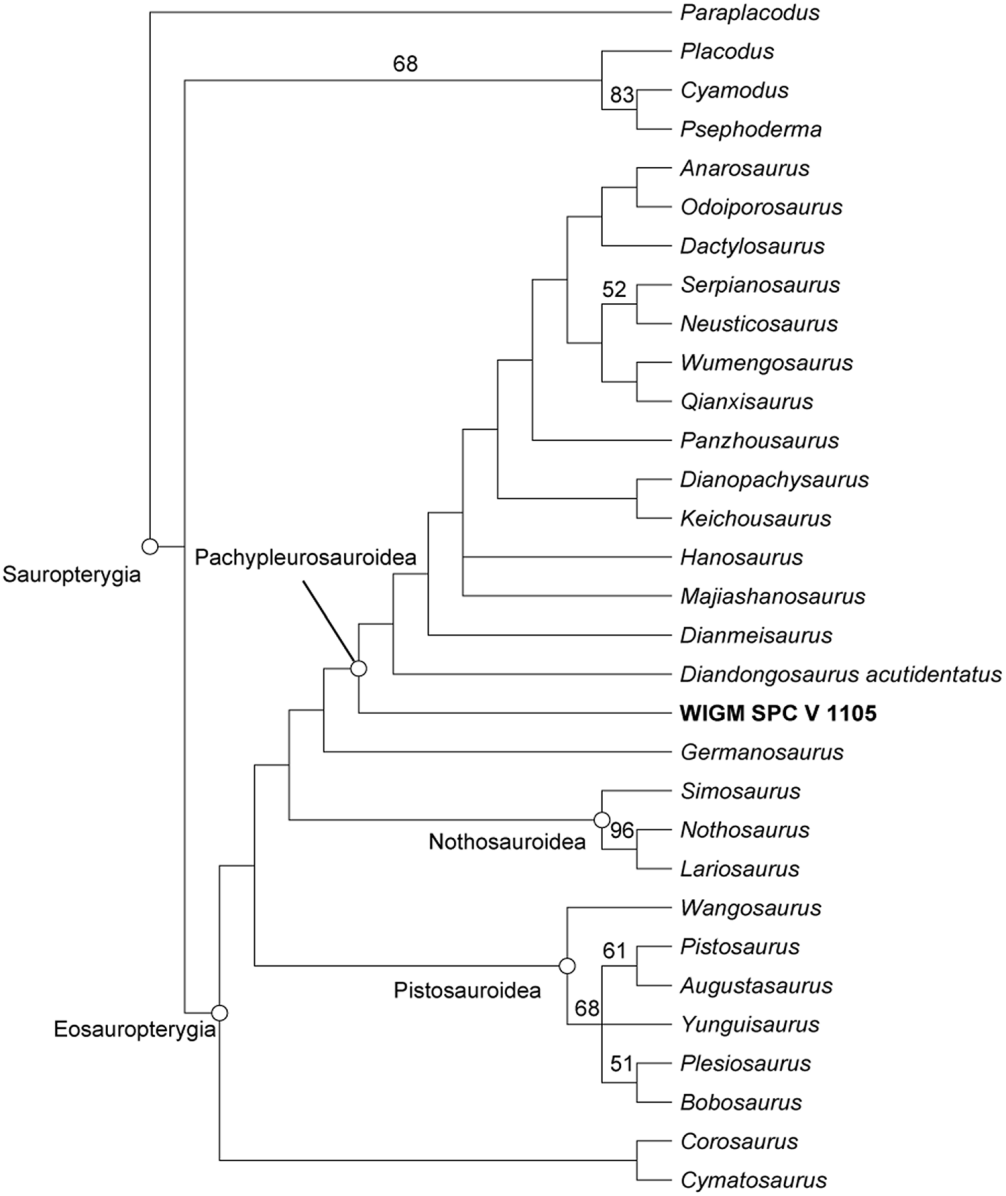
Strict consensus tree of four most parsimonious tree (TL = 485 steps, CI = 0.388, RI = 0.622), demonstrating the phylogenetic position of WIGM SPC V 1105. Bootstrap support values ≥ 50% (1000 replicates) are labelled. The figure is generated using Adobe Illustrator 2021 (https://www.adobe.com/products/illustrator.html).

We added WIGM SPC V 1105 to the cladistic matrix of Lin et al*.*^27^ and replicated their analytical methods in PAUP* version 4a169. Our cladistic analysis produced four most parsimonious trees (tree length = 485 steps, CI 0.388, RI 0.622). Strict consensus of these trees (Fig. 5) matches the result of former studies, in that *Diandongosaurus* share a close relationship with *Dianmeisaurus*^24^.

*Diandongosaurus* shows some similarities with *Keichousaurus* and *Dianopachysaurus*^18,19^, but many morphological differences exist. *Keichousaurus* and *Dianopachysaurus* have small tapering teeth^19,20^, while *Diandongosaurus* has serried long fang-shaped teeth. The supratemporal fenestra of *Diandongosaurus* is oval-shaped and larger than in the other two taxa considering the size of the orbit. The caudal ribs of *Dianopachysaurus* develop a tapering distal end, different from *Diandongosaurus*, whose caudal ribs have a flat distal end^17,20^.

*Diandongosaurus* also differs from other Triassic eosauropterygians. The strongly procumbent anterior teeth discriminate it from the pistosauroids, which have upright anterior teeth. The size of the supratemporal fenestra is noticeably larger than in *Qianxisaurus*^28^, while the characteristic tapering snout of *Wumengosaurus*^29^ differs from the blunt snout of *Diandongosaurus*. Its clavicle develops an anterior process, which does not exist in European pachypleurosaurs. *Diandongosaurus* has a smaller supratemporal fenestra than in *Lariosaurus* and *Nothosaurus*, in some species of which it is nearly twice the size of the orbit.

WIGM SPC V 1105 broadly resembles *D. acutidentatus* but differs in several features, including being considerably larger and the constricted snout of WIGM SPC V 1105 is a novelty in pachypleurosaur. These morphological distinctions between WIGM SPC V 1105 and *D. acutidentatus* could be regarded as evidence for establishing a new species. Alternatively, WIGM SPC V 1105 lacks the pterygoid opening in the two referred specimens (specimen NMNS-000933-F03498 and BGPDB-R0001) of *D. acutidentatus*^18,23^, and other differences, like the larger size and the rounded ends of humerus and femur, could have been caused by ontogenetic variation or even preservational issues. Based on previous documented specimens, interspecific variation of phalangeal formula exists in *D. acutidentatus*, as the pedal formular counts 2–3–4–5–4 in the holotype, but 2–3–4–6–4 in the referred specimen BGPDB-R0001^23^. In this case, WIGM SPC V 1105 could be an adult of *D. acutidentatus*. Given these considerations, we assigned WIGM SPC V 1105 as a conformis (cf.) of *D. acutidentatus*.

## Discussion

**Figure 6:**
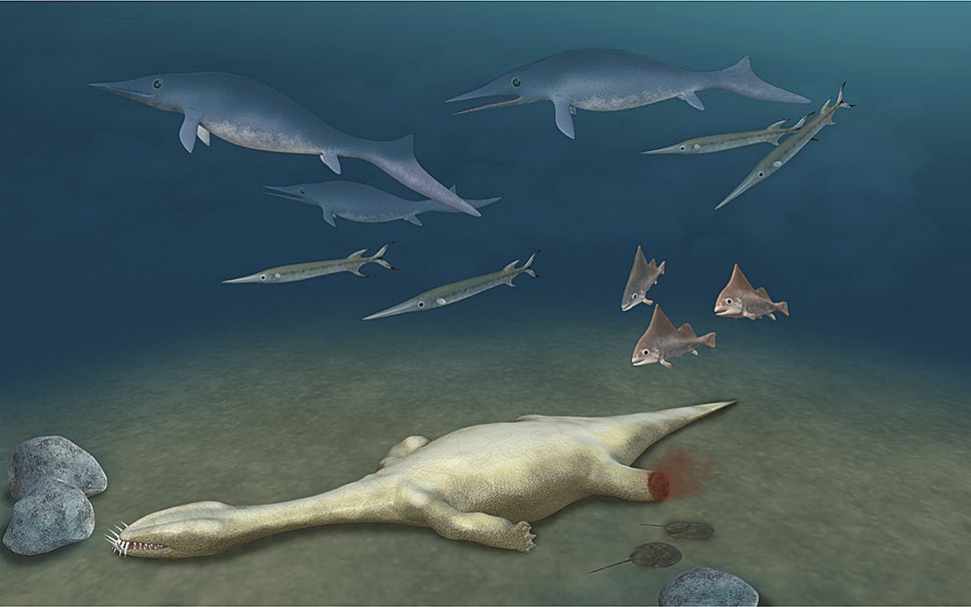
Artist’s restoration of WIGM SPC V 1105 attacked by a predator and lying dead. Illustrator: Tinglu Yang.

The skeleton of WIGM SPC V 1105 shows an amputated right hind limb (Figs. 1, 3c,d) which suggests an attack from an apex predator (Fig. 6). There are three lines of evidence: (1) the right femur is broken sharply across the middle and no trace of the distal half of the paddle can be found; (2) the remainder of the skeleton is well articulated and shows no sign of postmortem disturbance by currents; (3) there are two potential predators in the Luoping Biota: the nothosauroid *Nothosaurus zhangi*, whose right mandibular ramus is about 65 cm long^9^, and the archosaur *Qianosuchus mixtus*, over 3 m in length and equipped with dagger-like serrated teeth^30^. Further, WIGM SPC V 1105 was probably not an agile fast swimmer, as shown by its large body size, serried thick gastralia, and oblate pectoral girdle.

The unnaturally twisted digit 5 of the manual phalanges seems to be additional evidence for predatory action, especially when it is noted that the other phalangeal digits as well as the posteriormost caudal vertebrae are preserved in their original place, indicating a low-energy environment. However, taphonomic factors cannot be excluded, given that no bite marks have been found on the manual phalanges.

This is in line with the species richness of the Luoping Biota, including sauropterygians, ichthyosaurs, saurosphargids and protorosaurs as top predators^8^. The seven sauropterygians from Luoping (Table 2) were all predators, except the unexpected herbivorous marine reptile *Atopodentatus unicus*^31,32^. Further predators include two ichthyosaurs, *Mixosaurus* cf. *panxianensis*^33^ and *Phalarodon atavus*^34^, the turtle-like saurosphargids *Sinosaurosphargis yunguiensis*^35^ and *Largocephalosaurus polycarpon*^36^, as well as the protorosaur *Dinocephalosaurus orientalis*^37^ and the archosaur *Qianosuchus mixtus*^30^ from the nearby Panxian Biota^9^. The non-reptile fossil groups, such as arthropods, molluscs, echinoderms, brachiopods, fishes and plants, are rather diversified as well^8,10^.

**Table 2:** Marine reptile fossils of the Luoping Biota.

Taxa	Species	References
Archosaur	cf. *Qianosuchus*	Liu et al. (2014)^9^
Ichthyosaur	*Mixosaurus* cf. *panxianensis*	Liu et al. (2011)^19^
Ichthyosaur	*Phalarodon atavus*	Liu et al. (2013)^34^
Protorosaur	*Dinocephalosaurus* cf. *orientalis*	Liu et al. (2014)^9^
? Sauropterygian	*Atopodentatus unicus*	Cheng et al. (2014)^31^, Li et al. (2016)^32^
Sauropterygian	*Dawazisaurus Brevis*	Cheng et al. (2016)^38^
Sauropterygian	*Diandongosaurus acutidentatus*	Shang et al. (2011)^17^, Sato et al. (2014)^18^
Sauropterygian	*Dianmeisaurus gracilis*	Shang et al. (2015, 2017)^24,25^
Sauropterygian	*Dianopachysaurus dingi*	Liu et al. (2011)^33^
Sauropterygian	*Diandongosaurus* cf. *acutidentatus*	This study
Sauropterygian	*Nothosaurus zhangi*	Liu et al. (2014)^9^
Sauropterygian	*Lariosaurus* sp.	Liu et al. (2014)^9^
Saurosphargid	*Largocephalosaurus polycarpon*	Cheng et al. (2012)^28^, Li et al. (2014)^39^
Saurosphargid	*Sinosaurosphargis yunguiensis*	Li et al. (2011)^35^

More widely, the predatory marine reptiles were major new faunal components. There is no evidence that any of these groups existed in the Permian when the predators were primarily sharks and some bony fishes. The acceleration of life in the ‘modern fauna’ of the Triassic^40^ was marked by rich new faunas of invertebrates and fishes, with new clades of osteichthyans (neopterygians) and sharks (neoselachians). Among the reptiles were ichthyosaurs, placodonts and eosauropterygians, all of which emerged in the Olenekian (late Early Triassic), within 5 Myr after the PTME. Ichthyosaurs emerged as initially small, serpentine swimmers, and diversified enormously in the Middle Triassic, some of them reaching huge size in the Late Triassic. Placodonts, with their specialised mollusc-crushing dentitions, flourished through the Middle and Late Triassic. Among eosauropterygians, Pachypleurosauria first appeared in the Early Triassic with *Keichousaurus yuananensis*^41^, reached a diversity peak in the Middle Triassic with especially diverse records from southern China and Europe, and diminished and disappeared in the Late Triassic^20^. Nothosauroidea are mainly represented by *Nothosaurus* and *Lariosaurus*¸ with a first occurrence of the species *Lariosaurus sanxiaensis* in the Early Triassic^42^. Maximum diversity of over 20 species was reached in the Middle Triassic^9,43^, with a last record being *Paludidraco multidentatus* from the Carnian of Spain^44^ Pistosauroidea, like *Augustasaurus*^45^ and *Cymatosaurus*^46^, are found in the Middle Triassic, and the clade survived into the Jurassic and Cretaceous as plesiosaurs and pliosaurs^47^. These Triassic eosauropterygians ranged in size from pachypleurosaurs like *Keichousaurus* at 5 cm long^48^ to the nothosauroid *Nothosaurus* at 5–7 m^9^.

Such a developed trophic structure reflects a fully recovery after the PTME^3^, and the early stage of the MMR^2^. An early start for the MMR was already suggested^4^, based on high levels of boring predation marks on bivalve shells from the Early Jurassic. Such boreholes, evidence of the new gastropod predation modes, were reported also from the Triassic^49^. Strong external shell sculptures, interpreted as an antipredatory feature, and typical of the MMR^7^, are also reported in Triassic gastropods and bivalves^50^. This all suggests that the MMR began in the Triassic, and the likelihood is that this early start is associated with two consequences of the PTME. First is that the extinction cleared ecospace and allowed new taxa to dominate Triassic ecosystems, and these new taxa established new, faster life modes and arms races than seen in the Palaeozoic. Second are the more immediate aspects of the turmoil of post-PTME seas, when harsh environmental conditions interfered with the recovery and forced some strong ecological interactions. During the Early and Middle Triassic, new clades with their new adaptations emerged, both new antipredatory strategies such as thickened shells and cementation in oysters and mussels, snap escape swimming by scallops, motile crinoids, prominent sculpture in gastropods and bivalves, and deep burrowing by many taxa, as well as the new hunting modes, including shell snipping by malacostracans, hole boring using chemical and mechanical means by gastropods, and durophagy by diverse fishes and reptiles.

The evidence of predation shown by our specimen of WIGM SPC V 1105 fits with the wider evidence for predator–prey arms races in the Triassic. Though the European pachypleurosaur *Neusticosaurus edwardsii* from the Middle Triassic of Monte San Giorgio, reaches body sizes of 120 cm^51^, the pachypleurosaurs were small eosauropterygians in general, many less than 50 cm long, like *K. hui* and *Neusticosaurus peyeri*^48,52^. Thus, WIGM SPC V 1105 is relatively large for this group. Pachypleurosaurians were small enough to be eaten by fishes: specimens have been reported from the Middle Triassic of Monte San Giorgio as stomach contents of a 32-cm-long *Saurichthys* and within coprolites^53,54^. Further, a specimen of the hupehsuchian *Eohupehsuchus brevicollis* from the Nanzhang-Yuan’an Biota shows unusual preservation of its left forelimb, in which nearly half the manus was ripped off before burial, suggesting predation^55^. Yet these are relatively small marine reptiles feeding at low trophic levels^56^. Direct fossil evidence of predation on medium-to-large-sized carnivorous marine reptiles has been reported in mosasaurs associated with shark teeth in the Cretaceous^57^, but such finds are limited in the Triassic. The protorosaur *Macrocnemus* from the Middle Triassic of the eastern Swiss Alps could have been preyed upon by some marine predators, but it was probably a land-coastal reptiles rather than a marine predator^54,58^. Nevertheless, its relative *Tanystropheus* with a severed neck reported from the same place provide indirect evidence of predation^59^. A more direct case is a 5 m-long *Guizhouichthyosaurus* from the Xingyi Biota of the Middle Triassic, found with a skeleton of a thalattosaur in its stomach^60^. Such hypercarnivorous predation is relatively rare in the modern ocean, and even though apex predators like leopard seals and killer whales sometimes consume other tetrapods, they generally feed on low trophic level prey at times^1,61,62^.

## Material and methods

### Specimen

The specimen is WIGM (Wuhan Institute of Geology and Mineral Resources) SPC V 1105, a nearly complete skeleton accessioned in Wuhan Centre of China Geological Survey (WGSG) in Wuhan, Hubei Province, China. It was collected from Member II of the Guanling Formation at the quarry near Daaozi village, Luoping, north-eastern Yunnan Province, China. Surplus rock was removed from around the bones using fine needles and a mechanical dental drill. We used a binocular microscope, Olympus SZ61, in the preparation and observation of the specimen.

### Phylogenetic analysis

Cladistic analysis of relationships of the new specimen was conducted using the taxon-character data matrix in Lin et al*.*^27^, with added codings of the new specimen. The matrix consists of 31 taxa with 148 osteological characters defined by Lin et al*.*^22^, and excludes the two most fragmentary taxa (“Online Supplement”). The cladistic analysis was performed using PAUP* 4a169 (Ref.^63^), implementing a heuristic search with all characters unordered and equally weighted, followed by a ‘fast’ stepwise-addition search to calculate the bootstrap values with 1000 replications.

## Supplementary Information

Supplementary Information.

## Data Availability

The datasets generated and/or analysed during the current study can be found via the Dryad Digital Repository at: https://datadryad.org/XXX.
