# Supplementary material for: An injured pachypleurosaur (Diapsida: Sauropterygia) from the Middle Triassic Luoping Biota indicating predation pressure in the Mesozoic
Source: Sci Rep. 2021 Nov 8;11:21818. doi: 10.1038/s41598-021-01309-z (PMC8575933; doi:10.1038/s41598-021-01309-z)
Supplement: Supplementary file 1 — Supplementary Information. [file 41598_2021_1309_MOESM1_ESM.pdf]

Supplementary materials for

An injured pachypleurosaur (Diapsida: Sauropterygia) from the Middle Triassic Luoping Biota indicating predation pressure in the Mesozoic

Qiling Liu[1,3], Tinglu Yang[2], Long Cheng[3]*, Michael J. Benton[4], Benjamin C. Moon[4], Chunbo Yan[3], Zhihui An[3], Li Tian[1]*

[1]State Key Laboratory of Biogeology and Environmental Geology, China University of Geosciences, Wuhan, 430078, P. R. China.
[2]School of Earth Science, East China University of Technology, Nanchang, Jiangxi Province, 330013, P. R. China.
[3]Hubei Key Laboratory of Paleontology and Geological Environment Evolution, Wuhan Centre of China Geological Survey, Wuhan, Hubei, 430023, P. R. China.
[4] School of Earth Sciences, Life Sciences Building, Tyndall Avenue, University of Bristol, Bristol BS8 1TQ, UK.
*Correspondences to emails: chengl@mail.cgs.gov.cn (L.C.); tianlibgeg@cug.edu.cn (L.T.)

Data matrix for phylogenetic analysis

Our cladistic analysis of relationships of the new species was conducted using the taxon-character data matrix in Lin et al. [1], and adding the codings of the new specimen to the original matrix. The matrix consists of 31 known taxa with 148 osteological characters defined by Lin et al. [1]. The data matrix is shown in Nexsus format and the code of the new specimen is shown below.

WIGM SPC V 1105

0 0 1 0 1 1 0 1 0 0 0 0 ? 0 1 0 0 0 1 1 0 1 1
0 0 1 0 0 0 0 1 1 1 0 3 0 2 1 1 0 0 0 ? ? 1
? 0 0 0 1 1 1 0 ? 0 1 0 0 2 0 ? 0 ? 0 2 ? 1
0 0 1 1 0 ? 0 2 ? 1 ? ? ? ? 0 0 0 ? ? ? ? ?
? ? 0 1 0 0 0 0 0 0 1 1 0 ? 2 0 1 1 ? 2 0 0
0 1 ? 1 1 1 0 2 0 0 0 0 0 0 ? 1 1 1 1 1 1 1
2 0 1 1 1 1 0 0 0 0 ? 1 1 0 ?

Literature cited

1. Lin, W.-B. *et al. Panzhousaurus Rotundirostris* Jiang et al., 2019 (Diapsida: Sauropterygia) and the Recovery of the Monophyly of Pachypleurosauridae. *J. Vert. Paleontol.* e1901730; 10.1080/02724634.2021.1901730 (2021).
